# MAPK regulates secondary metabolism and abiotic stress in horticultural and medicinal plants

**DOI:** 10.1093/hr/uhaf350

**Published:** 2025-12-17

**Authors:** Shuanglu Liu, Minghui Xing, Xiaojian Yin

**Affiliations:** Key Laboratory of Soybean Molecular Design Breeding, State Key Laboratory of Black Soils Conservation and Utilization, Jilin Da'an Agro-Ecosystem National Observation and Research Station, Northeast Institute of Geography and Agroecology, Chinese Academy of Sciences, Changchun 130102, China; University of Chinese Academy of Sciences, Beijing 100049, China; Key Laboratory of Soybean Molecular Design Breeding, State Key Laboratory of Black Soils Conservation and Utilization, Jilin Da'an Agro-Ecosystem National Observation and Research Station, Northeast Institute of Geography and Agroecology, Chinese Academy of Sciences, Changchun 130102, China; Key Laboratory of Soybean Molecular Design Breeding, State Key Laboratory of Black Soils Conservation and Utilization, Jilin Da'an Agro-Ecosystem National Observation and Research Station, Northeast Institute of Geography and Agroecology, Chinese Academy of Sciences, Changchun 130102, China

## Abstract

Horticultural and medicinal plants are important for their economic and pharmacological value; however, their quality traits are severely affected by abiotic stresses. The mitogen-activated protein kinase (MAPK) cascade is an evolutionarily conserved signaling module that links abiotic stress signals to the regulation of plant quality traits. While the roles of MAPKs in growth, phytohormone signaling, and immunity are well established, a comprehensive review that integrates MAPK functions in abiotic stress responses and secondary metabolism, particularly in horticultural and medicinal plants, is still lacking. In this review, we systematically summarize (i) the composition, classification, and phylogenetic relationships of MAPKs in horticultural and medicinal plants; (ii) their mechanistic involvement in abiotic stress responses, particularly to salt, drought, and extreme temperatures; (iii) recent advances in understanding how MAPK-mediated signaling governs secondary metabolite accumulation; and (iv) a unified framework that presents MAPKs as a key bridge between stress responses and metabolic reprogramming. These insights provide a foundation for MAPK-targeted breeding and engineering strategies that enhance stress tolerance and improve quality traits in horticultural and medicinal plants through precise pathway manipulation.

## Introduction

Horticultural and medicinal plants are high-value pillars of global agriculture; they sustain food supply and nutritional diversity through providing medicinal and functional compounds [1–3]. Their flavor, color, aroma, texture, and pharmacological activity depend on the fine coordination of specialized metabolic pathways, yet abiotic stresses such as drought, salt, and extreme temperatures often compromise yield and quality [3–5]. To cope with these challenges, plants have evolved tiered and efficient defense systems that can be categorized as tolerance, avoidance, escape, and recovery strategies [6–8]. Upon stress perception, plants rapidly trigger Ca^2+^ spikes and phosphorylation cascades, followed by membrane lipid/cell wall remodeling, accumulation of osmolytes and antioxidants, dynamic control of reactive oxygen species (ROS)/reactive nitrogen species (RNS), and transcriptional reprogramming, which ultimately culminate in adjustments in water balance, reconfiguration of photosynthesis and energy metabolism, and recalibration of growth, reproduction, and senescence [9–11].

Secondary metabolites carry dual value as determinants of product quality and as stress protectants [12]. Secondary metabolites are nonessential small molecules with pronounced species and spatiotemporal specificity, broadly classified by biosynthetic origin into three groups: alkaloids, typically nitrogenous and arising via shikimate- and 2-C-methyl-D-erythritol 4-phosphate (MEP)–related routes; terpenoids [2], derived from the C5 precursors isopentenyl pyrophosphate and dimethylallyl pyrophosphate (IPP and DMAPP) through the plastidial MEP pathway and the cytosolic mevalonate (MVA) pathway [12]; and phenylpropanoids and their derivatives, expanded by multistep modifications of a shikimate–phenylpropanoid core. Under adverse conditions, secondary metabolites enhance adaptation by inhibiting microbes and herbivores, scavenging ROS [13], fortifying cell walls, and modulating signaling (e.g. nicotine, camalexin, flavonoids), and they also provide key medicines for humans (e.g. artemisinin, paclitaxel, tanshinones, salvianolic acids) [2, 14]. Given that the potential metabolome may exceed a million entities and that accu mulation is multilayer-regulated, simple ‘single-enzyme, single-pathway’ models are inadequate to explain this complexity.

Among post-translational modifications (PTMs), protein phosphorylation serves as a pivotal mechanism that translates external stimuli into intracellular responses [15]. The canonical mitogen-activated protein kinase (MAPK) cascade, a highly conserved signaling module in eukaryotes, operates hierarchically: receptor activation triggers an MAPK kinase kinase (MAPKKK), which phosphorylates the conserved S/T–XXXXX–S/T motif of an MAPK kinase (MAPKK) [16]; the activated MAPKK then phosphorylates the Thr-any-Tyr (TXY) motif within an MAPK, thereby activating the terminal kinase [16, 17]. Based on the conserved TXY activation loop motif targeted by MAPKKs, plant MAPKs are divided into two major subfamilies: one carrying the Thr-Glu-Tyr (TEY) motif and the other the Thr-Asp-Tyr (TDY) motif (Group D) [16, 18]. The TEY-type subfamily can be further subdivided into Groups A–C [16–18], distinguished by specific structural and sequence characteristics. Once activated, MAPKs phosphorylate diverse downstream targets, including transcription factors (TFs), structural enzymes, and other regulatory proteins, thus relaying and amplifying extracellular cues with high precision [19, 20]. Through these phosphorylation events, MAPKs orchestrate essential processes such as plant growth and development, phytohormone signaling, responses to biotic and abiotic stresses, and the regulation of secondary metabolism [21–25].

In recent years, the roles of MAPKs in abiotic stress responses and secondary metabolite biosynthesis in horticultural and medicinal plants have attracted increasing attention. However, a comprehensive review of how MAPKs coordinate abiotic stress responses and reprogram secondary metabolism in these plants is still lacking; prior work often treats stress adaptation and metabolic control separately, with limited cross-species comparisons and module-level summaries. In this review, we present an integrated overview of: (i) the composition, classification, and phylogenetic relationships of MAPKs in horticultural and medicinal plants; (ii) the response and roles of MAPK cascades in mediating tolerance to salt, drought, and extreme temperatures (Tables 2 and 3); (iii) their functions and molecular mechanisms in regulating secondary-metabolite accumulation, including phenylpropanoids (anthocyanins, lignin, salvianolic acids, sakuranetin, forsythin), terpenoids (tanshinones, crocins, diterpenoid, linalool), alkaloids (monoterpenoid indole alkaloids, nicotine), and other defensive metabolites (phytoalexins, glucosinolates) (Table 4); and (iv) a unified framework positioning the MAPKs as a key bridge between stress resilience and metabolite accumulation. These insights provide actionable targets and strategies for MAPK-informed molecular breeding and metabolic engineering to jointly improve stress adaptability and quality traits.

## Composition, classification, and phylogenetic relationships of MAPKs in model, horticultural, and medicinal plants

MAPKs represent one of the most conserved signaling modules in plants, translating extracellular cues into intracellular responses that control growth, development, and stress adaptation. Understanding their composition, classification, and evolutionary relationships across model, horticultural, and medicinal plants is therefore essential to uncover how conserved MAPK signaling frameworks have diversified to meet lineage-specific physiological demands. Despite extensive studies in *Arabidopsis thaliana* [26–30] and *Oryza sativa* [31–36], the organization and evolutionary patterns of *MAPKs* in many horticultural and medicinal plants remain poorly understood. In particular, whether these species possess lineage-specific MAPK subfamilies or motif innovations has not been systematically evaluated.

In our comparative genomic analysis of 21 representative species, we identified 10–26 MAPK members per genome (Table 1; Table S1). Across species, the D group consistently constituted the largest subclass, while Group C was often reduced or absent. Most MAPKs contained conserved TEY or TDY activation motifs, forming a stable structural backbone, whereas rare motif variants such as Thr-Asn-Tyr (TNY) in *Limonium bicolor* [37] and Met-Glu-Tyr (MEY) in *Solanum melongena* [38] imply clade-specific functional divergence.

**Table 1:** Comparative landscape of MAPK family size, TXY motifs, and A–E subgroups across model, horticultural and medicinal plants

**ID**	**Species**	**MAPK count**	**Subgroups**					**TXY Motifs**
			**A**	**B**	**C**	**D**	**E**	
1	*Actinidia chinensis*	18	4	5	3	3	3	TEY/TDY
2	*Aquilegia coerulea*	11	5	3	0	3	0	TEY/TDY
3	*A. thaliana*	20	3	5	4	8	0	TEY/TDY
4	*Camellia sinensis*	21	6	4	3	8	0	TEY/TDY
5	*C. illinoinensis*	18	4	4	3	7	0	TEY/TDY
6	*C. morifolium*	11	3	3	1	4	0	TEY/TDY
7	*C. sativus*	14	2	3	1	8	0	TEY/TDY
8	*Daucus carota*	17	6	3	0	8	0	TEY/TDY
9	*Eucommia ulmoides*	13	2	4	2	5	0	TEY/TDY
10	*Fagopyrum tataricum*	16	2	4	1	9	0	TEY/TDY
11	*Fragaria vesca*	12	3	2	2	5	0	TEY/TDY
12	*L. bicolor*	20	3	6	4	7	0	TEY/TDY/TNY
13	*M. domestica*	26	5	6	5	10	0	TEY/TDY
14	*M. notabilis*	10	2	3	2	1	2	TEY/TDY
15	*N. nucifera*	15	4	4	1	6	0	TEY/TDY
16	*O. sativa*	17	2	2	2	11	0	TEY/TDY/MEY
17	*S. miltiorrhiza*	18	3	5	2	8	0	TEY/TDY
18	*S. lycopersicum*	16	3	4	2	7	0	TEY/TDY/MEY
19	*S. melongena*	16	3	4	1	8	0	TEY/TDY/MEY
20	*V. vinifera*	14	2	3	2	5	2	TEY/TDY
21	*Ziziphus jujuba*	10	2	2	1	5	0	TEY/TDY

Notes: MEY (Met-Glu-Tyr); TXY (Thr-Any amino acid-Tyr); TDY (Thr-Asp-Tyr); TEY (Thr-Glu-Tyr); TNY (Thr-Asn-Tyr). Groups A–E denote MAPK subgroups according to canonical plant MAPK classification.

**Figure 1:**
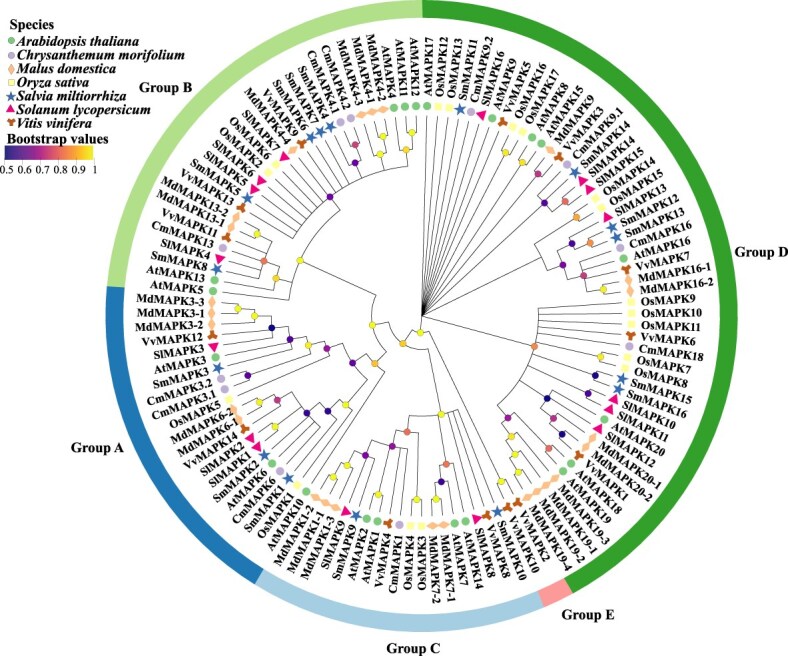
Phylogenetic analysis of MAPKs in model, horticultural, and medicinal plants. Twenty *AtMAPKs* from *A. thaliana*, 11 *CmMAPKs* from *C. morifolium*, 26 *MdMAPKs* from *M. domestica*, 17 *OsMAPKs* from *O. sativa*, 18 *SmMAPKs* from *S. miltiorrhiza*, 16 *SlMAPKs* from *S. lycopersicum*, and 14 *VvMAPKs* from *V. vinifera* were analyzed. An NJ tree was constructed using MEGA 11 with 1000 bootstrap replicates. Node color gradients indicate bootstrap support values (0.5–1.0, reflecting branch reliability); and connecting lines illustrate phylogenetic relationships among *MAPKs*.

A neighbor-joining (NJ) tree was constructed using MEGA 11 with 1000 bootstrap replicates [39]. Phylogenetic reconstruction of seven representative species—*A. thaliana* [26], *Chrysanthemum morifolium* [40], *Malus domestica* [41], *O. sativa* [31], *Salvia miltiorrhiza* [42], *Solanum lycopersicum* [43], and *Vitis vinifera* [44]—clustered all MAPKs into five well-supported groups (A–E) (Fig. 1). Groups A and D contained the most members; Group A was conserved across all lineages, while Group D exhibited marked diversification in *S. miltiorrhiza*, *M. domestica*, and *O. sativa*, suggesting potential neofunctionalization.

Together, these findings reveal a conserved TEY/TDY-based MAPK backbone combined with lineage-specific expansions and occasional motif innovations, reflecting both evolutionary stability and adaptive diversification. Future research should focus on integrating functional and structural analyses to determine how MAPK diversification contributes to species-specific stress signaling and metabolic specialization in horticultural and medicinal plants.

## The response, function, and regulatory network of MAPK in abiotic stress

### MAPKs involved in abiotic stress response

MAPK cascades act as central hubs translating abiotic stress signals, such as drought, salt, and extreme temperature, into transcriptional and metabolic adjustments that sustain cellular homeostasis [8, 11, 45–47]. While the roles of MAPKs in stress signaling are well characterized in model plants like *A. thaliana* and *O. sativa*, their diversity and functional specialization in horticultural and medicinal plants remain less understood [26, 27, 29, 30, 35, 48–54]. Recent transcriptomic analyses across multiple species reveal that most *MAPK* genes respond dynamically to abiotic stresses, showing species- and organ-specific expression patterns (Table 2). In general, drought and salt trigger broad transcriptional activation, whereas temperature stress elicits more heterogeneous responses—high temperature inducing widespread upregulation and low temperature producing both activation and repression depending on the species. For instance, several MAPKs in *Carya illinoinensis* [55], *C. morifolium* [40], *Medicago sativa* [56], and *Morus notabilis* [57] were strongly induced under drought or salt conditions, reflecting a conserved stress-responsive module. Such pervasive responsiveness underscores MAPKs as critical mediators linking environmental cues to adaptive physiological remodeling. In summary, *MAPK* genes in horticultural and medicinal plants exhibit extensive yet differentiated transcriptional regulation under abiotic stress, suggesting lineage-specific specialization built upon a conserved signaling backbone. Future studies should move beyond expression profiling to integrate functional genomics and phosphoproteomic approaches, aiming to elucidate how specific MAPK modules coordinate crosstalk among Ca^2+^, ROS, and phytohormone pathways to fine-tune stress resilience [58, 59].

**Table 2:** Overview of MAPKs in abiotic stress responses across species

**Abiotic stress**	**Species**	**MAPK**	**Up/Down**	**Reference**
Drought	*C. illinoinensis*	*CiPawMAPK1/3–1/13*	Up	[55]
	*C. morifolium*	*CmMAPK4.1/4.2/13*	Up	[40]
	*C. morifolium*	*CmMAPK9.2/16/18*	Down	[40]
	*F. tataricum*	*FtMAPK1/3*	Up	[60]
	*F. tataricum*	*FtMAPK4/9*	Down	[60]
	*F. vesca*	*FvMAPK5/8*	Up	[61]
	*Helianthus annuus*	Leaves: *HaMAPK3–2/11–1/14/1/6–2/19–1/18* Roots: *HaMAPK3–2/9–2/11–2/13–2/23–2*	Up	[62]
	*H. annuus*	Leaves: *HaMAPK13–2* Roots: *HaMAPK14*	Down	[62]
	*L. bicolor*	*LbMAPK10/18/19*	Up	[37]
	*L. bicolor*	*LbMAPK2/5/7/8/14/17/20*	Down	[37]
	*M. notabilis*	*MnMAPK3/4/6/7/8/9*	Up	[57]
	*M. notabilis*	*MnMAPK1/2*	Down	[57]
	*M. sativa*	*MsMAPK1/5/7/33/71/73*	Up	[56]
	*M. sativa*	*MsMAPK60/63/64*	Down	[56]
Salt	*A. chinensis*	*AcMAPK4/5/9/10/12/13/17*	Up	[63]
	*C. morifolium*	*CmMAPK3.2/13*	Up	[40]
	*F. tataricum*	*FtMAPK1/3/4/9*	Down	[60]
	*F. vesca*	*FvMAPK5/9/10/11/12*	Up	[61]
	*Glycyrrhiza uralensis*	*GuMAPK5/7/9/16/20–2/20–3*	Up	[64]
	*H. annuus*	Leaves: *HaMAPK11–1* Roots: *HaMAPK2/6–1/14/23–2/17*	Up	[62]
	*H. annuus*	Leaves: *HaMAPK7/23–1* Roots: *HaMAPK9–2/13–2*	Down	[62]
	*L. bicolor*	*LbMAPK10/12/18*	Up	[37]
	*L. bicolor*	*LbMAPK13/16/20*	Down	[37]
	*M. notabilis*	*MnMAPK1/9/10*	Up	[57]
	*M. notabilis*	*MnMAPK3/4/7/8*	Down	[57]
	*M. sativa*	*MsMAPK1/3/5/7/28/33/65/70/71/75*	Up	[56]
	*S. melongena*	*SmMAPK4.1*	Up	[38]
Heat	*A. chinensis*	*AcMAPK1/5/10/11/14/15/16/17/18*	Up	[63]
	*C. morifolium*	*CmMAPK1/9.1/9.2/16/18*	Down	[40]
	*F. vesca*	*FvMAPK3*	Up	[61]
	*M. notabilis*	*MnMAPK1/5/6/9*	up	[57]
	*M. notabilis*	*MnMAPK2/3/8/10*	Down	[57]
Cold	*A. chinensis*	*AcMAPK4/5/9/10/11/12*	Up	[63]
	*A. chinensis*	*AcMAPK2/6/7/13/18*	Down	[63]
	*C. morifolium*	*CmMAPK1/3.1/3.2/4.2/6/9.1/9.2/13/16/18*	Up	[40]
	*M. notabilis*	*MnMAPK1/5*	Up	[57]
	*M. notabilis*	*MnMAPK2/3/4/6/7/8*	Down	[57]
	*M. sativa*	*MsMAPK5/7/50/53/67/70/73/75/78*	Up	[56]
	*M. sativa*	*MsMAPK51/60*	Down	[56]
	*S. melongena*	*SmMAPK4.1*	Up	[38]

### MAPK-mediated regulation of phytohormone signaling and ROS homeostasis enhances plant drought tolerance

Drought represents one of the most pervasive abiotic stresses that severely constrain plant productivity and survival [45, 49]. In horticultural and medicinal plants, which are often cultivated under variable environments, drought not only restricts biomass accumulation but also affects secondary metabolite profiles critical for product quality [6, 11]. Understanding the molecular mechanisms that mediate drought tolerance is thus fundamental for improving both yield and quality. Among various signaling modules, MAPK cascades serve as evolutionarily conserved sensors and transducers that translate drought-induced cellular perturbations into adaptive transcriptional and metabolic responses. Although MAPK cascades have been extensively characterized in model species, the specific molecular circuits by which they integrate phytohormone signaling, ROS homeostasis, and secondary metabolism in horticultural and medicinal plants remain poorly understood. Particularly, it is unclear how different MAPK modules confer organ- or species-specific drought tolerance and how they connect early stress perception to downstream metabolic adaptation [11].

**Figure 2:**
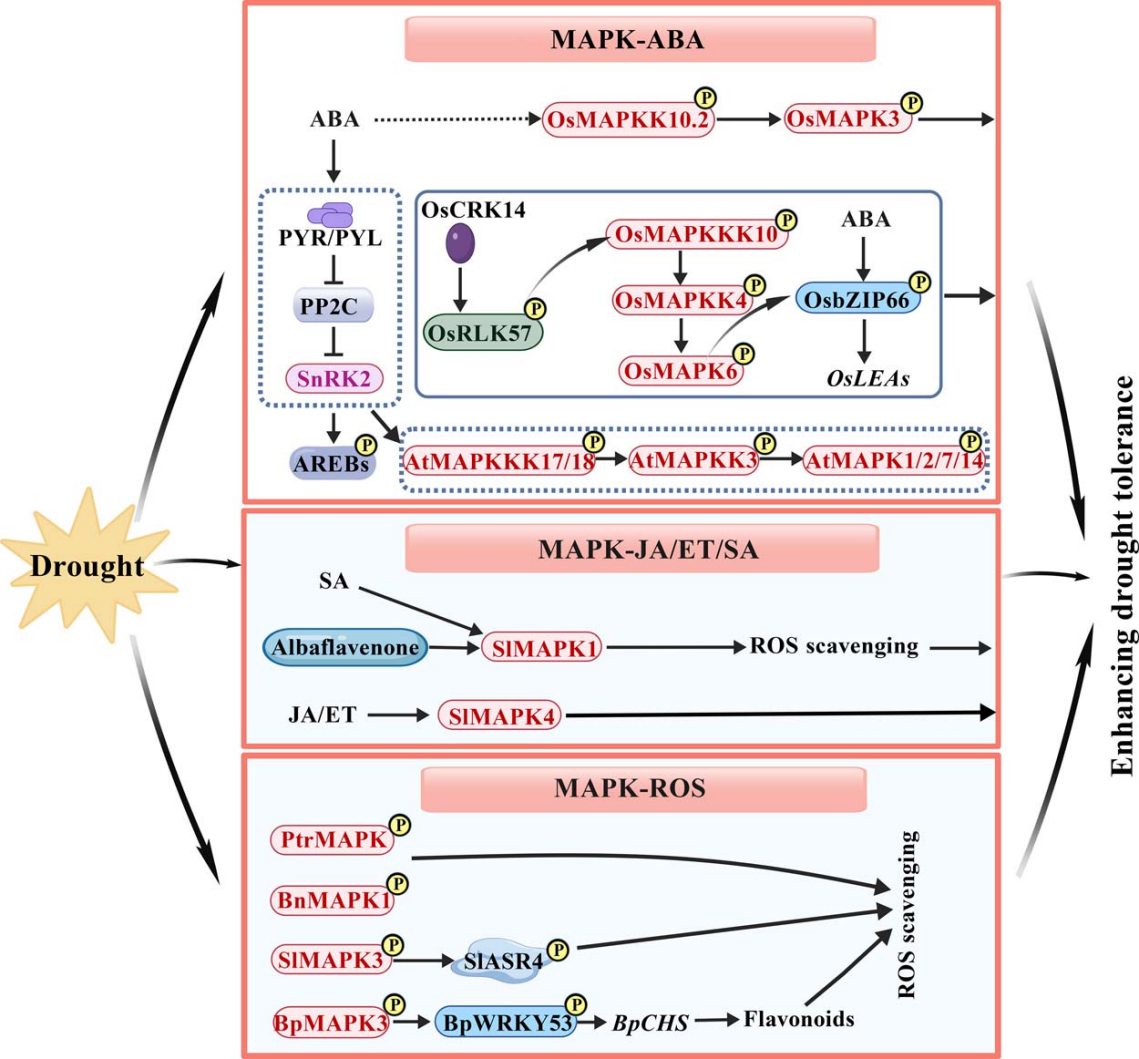
MAPK is involved in drought stress responses in horticultural and medicinal plants. Drought activates multiple MAPK cascades that integrate ABA, JA, and SA signaling with ROS homeostasis to enhance stress resilience. In the ABA pathway, OsMAPKK10.2–OsMAPK3 and OsCRK14–OsRLCK57–OsMAPKKK10–OsMAPKK4–OsMAPK6 stabilize OsbZIP66 and induce *OsLEAs*, while AtMAPKKK17/18–AtMAPKK3–AtMAPK1/2/7/14 represents a conserved ABA-dependent module. In the JA/ET/SA branch, SlMAPK1 and SlMAPK4 coordinate SA-, JA-, and ET-mediated ROS scavenging. In the ROS-regulatory branch, SlMAPK3 interacts with SlASR4 to remove ROS, and BpMAPK3 phosphorylates BpWRKY53 to activate *BpCHS* and flavonoid biosynthesis. Similarly, MAPK activation in *P. trifoliata* and *B. napus* enhances antioxidant enzyme activity. The figure is created with BioGDP.com [77]. ABA, abscisic acid; ET, ethylene; AREBs, ABA-RESPONSIVE ELEMENT-BINDING PROTEINS; CHS, CHALCONE SYNTHASE; JA, jasmonic acid; MAPK, MITOGEN-ACTIVATED PROTEIN KINASE; MAPKK, MAPK KINASE; MAPKKK, MAPK KINASE KINASE; SA, salicylic acid; ROS, reactive oxygen species; SnRK2, SNF1-RELATED PROTEIN KINASE 2; OsCRK14, CYSTEINE-RICH RECEPTOR-LIKE KINASE 14; OsRLCK57, RECEPTOR-LIKE CYTOPLASMIC KINASE 57; OsbZIP66, BASIC LEUCINE-ZIPPER TRANSCRIPTION FACTOR 66; OsLEAs, LATE EMBRYOGENESIS ABUNDANT PROTEINS; SlASR4, ABSCISIC ACID STRESS RIPENING PROTEIN 4.

Recent studies across diverse species highlight that MAPKs act as central nodes linking phytohormone and redox signaling during drought (Fig. 2; Table 3). In Arabidopsis, the AtMAPKKK17/18–AtMAPKK3–AtMAPK1/2/7/14 cascade constitutes a core abscisic acid (ABA)-dependent pathway [65–67], where SNF1-RELATED PROTEIN KINASE 2 (SnRK2) activates the MAPK cascade and the TF ABSCISIC ACID-RESPONSIVE ELEMENT-BINDING PROTEIN 1 (AtAREB1) to amplify ABA responses [67]. A similar ABA-dependent cascade, OsMAPKK10.2–OsMAPK3, operates in rice (*O. sativa*) [68]. Furthermore, the CYSTEINE-RICH RECEPTOR-LIKE KINASE 14 (OsCRK14)–RECEPTOR-LIKE CYTOPLASMIC KINASE 57 (OsRLCK57)–OsMAPKKK10–OsMAPKK4–OsMAPK6 module enhances the stability of the ABA-responsive BASIC LEUCINE-ZIPPER TRANSCRIPTION FACTOR 66 (OsbZIP66) [35], thereby strengthening drought tolerance.

**Table 3:** Functions and mechanisms of MAPKs in plant abiotic stress

**Abiotic stresses**	**Species**	**MAPKs**	**Substrates/Pathways**	**Reference**
Drought	*A. thaliana*	AtMAPK1/2/7/14	AtMAPKKK17/18–MAPKK3–MAPK1/2/7/14	[65–67]
	*B. platyphylla*	BpMAPK3	BpWRKY53	[72]
	*B. napus*	BnMAPK1	Enhance antioxidant enzyme activity	[74]
	*O. sativa*	OsMAPK6	OsbZIP66	[35]
	*O. sativa*	OsMAPK3/6	OsMAPKK10.2-MAPK3/6	[68]
	*P. trifoliata*	PtrMAPK	ROS	[73]
	*S. lycopersicum*	SlMAPK1	ROS	[69]
	*S. lycopersicum*	SlMAPK3	Enhance antioxidant responses	[71]
	*S. lycopersicum*	SlMAPK4	JA/ET	[70]
Salt	*A. thaliana*	AtMAPK4/6	AtMAPKK2–MAPK4/6	[79]
	*L. bicolor*	LbMAPKs	Salt gland development	[37]
	*M. domestica*	MdMAPK3	MdWRKY17	[83]
	*M. sativa*	MsMAPK3	MsNAC73	[23]
	*O. sativa*	OsMAPK3/6	OsCPK5/13-OsMAPK3/6	[51]
	*O. sativa*	OsMAPK4	OsIPA1	[53]
	*O. sativa*	OsMAPK5	OsSERF1	[80]
	*O. sativa*	OsMAPK3/6	OsSIT1-MAPK3/6	[59]
	*Z. mays*	ZmMAPK7	ZmWRKY104	[81]
	*Z. mays*	ZmMAPK3	ZmGRF1	[84]
Heat	*A. thaliana*	AtMAPK3/6	AtIAA8	[90]
	*C. annuum*	CaMAPK1	JA/SA/ABA-CaMAPK1 -HSFA2 /70–1	[86]
	*C. sativus*	CsMAPK4	CsVQ10	[85]
	*S. lycopersicum*	SlMAPK1	SlSPRH1	[88]
	*S. lycopersicum*	SlMAPK1	Photosynthesis	[87]
	*L. sativa*	LsMAPK4	Unidentified	[89]
Cold	*A. thaliana*	AtMAPK3/4/6	AtICE1	[91, 92]
	*I. batatas*	IbMAPK3/6	IbSPF1	[95]
	*O. sativa*	OsMAPK3	OsMAPKK6-MAPK3- bHLH002-TPP1	[33]
	*S. lycopersicum*	SlMAPK1/2	SlBBX17-HY5-CBF1	[93]
	*S. lycopersicum*	SlMAPK7	ROS	[94]

MAPK–phytohormone crosstalk extends beyond ABA. In tomato (*S. lycopersicum*), SlMAPK1 promotes ROS scavenging and enhances drought tolerance. Its activation by salicylic acid (SA) and microbial metabolite albaflavenone further illustrates a microbe-induced MAPK–SA signaling collaboration [69], while SlMAPK4 links jasmonic acid (JA) and ethylene (ET) pathways [70], reflecting an integrated defense network. MAPKs also fine-tune drought tolerance by modulating ROS homeostasis and secondary metabolism. In tomato, SlMAPK3 interacts with the ABSCISIC ACID STRESS RIPENING PROTEIN 4 (SlASR4) to scavenge ROS, thereby enhancing drought tolerance [71]. In birch (*Betula platyphylla*), BpMAPK3 activates the TF BpWRKY53 to upregulate flavonoid biosynthetic genes, thereby maintaining ROS balance [72]. In *Poncirus trifoliata* and *Brassica napus*, MAPK activation enhances antioxidant enzyme activities, thereby improving drought tolerance [73, 74]. In *S. miltiorrhiza*, transcriptomic analyses revealed enrichment of MAPK signaling, phenylpropanoid metabolism, and tanshinone biosynthesis under drought, suggesting a direct regulatory role of SmMAPKs in secondary metabolism [75, 76].

Collectively, these studies reveal that MAPK cascades function as dual regulators in plant drought responses, integrating early stress signaling with metabolic control. By coordinating ABA and JA signaling, regulating ROS-scavenging systems, and modulating secondary metabolism, MAPKs act as pivotal molecular switches that connect environmental perception to adaptive reprogramming. Future research should focus on dissecting MAPK-specific modules that underlie organ- or species-specific drought tolerance, identifying direct MAPK substrates through phosphoproteomics, and elucidating how MAPK-mediated phosphorylation reshapes phytohormone sensitivity and metabolic fluxes. Integrating omics, gene editing, and biochemical approaches in horticultural and medicinal plants will clarify how MAPK diversification contributes to their evolutionary and functional adaptation to drought.

### MAPK-mediated regulation of phytohormone signaling, ion homeostasis, and ROS detoxification enhances plant salt tolerance

**Figure 3:**
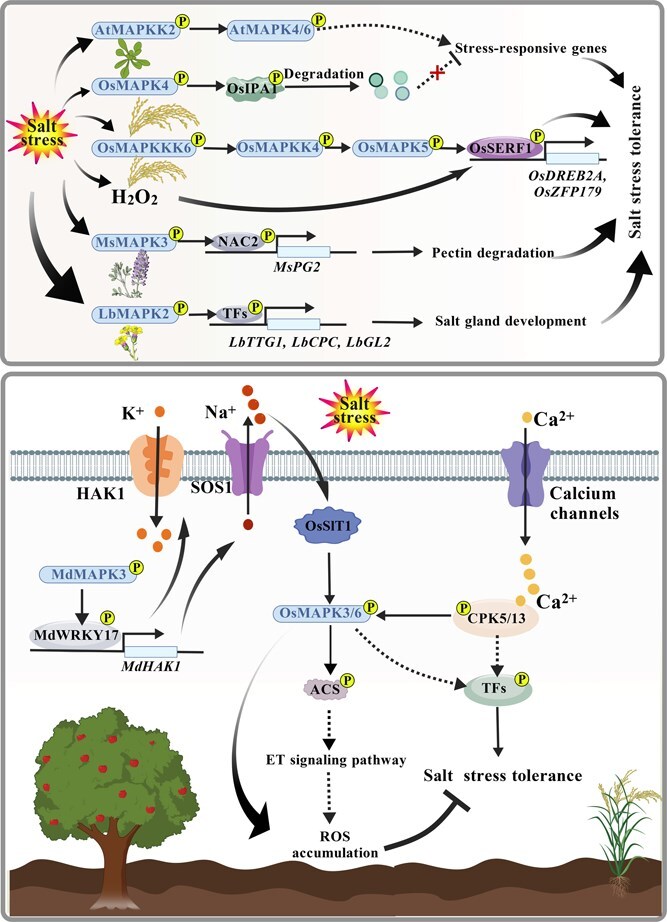
Salt stress signal transduction mediated by MAPK in plants. Salt stress activates diverse MAPK cascades that integrate hormonal signaling, ion balance, and ROS regulation to promote salt tolerance. In Arabidopsis, the AtMAPKK2–AtMAPK4/6 module activates stress-responsive genes. In rice, OsMAPK5 phosphorylates OsSERF1 to induce *OsDREB2A* and *OsZFP179*, while OsMAPK4 suppresses salt tolerance by targeting OsIPA1. OsSIT1 activates OsMAPK3/6 to regulate ethylene signaling and ROS levels, and OsCPK5/13 forms a Ca^2+^-dependent regulatory loop with MAPKs and TFs. In horticultural and medicinal plants, MsMAPK3–MsNAC2–MsPG2 modulates cell wall remodeling in *M. sativa*, LbMAPK2 promotes salt gland development in *L. bicolor*, and MdMAPK3–WRKY17–HAK1 maintains Na^+^/K^+^ homeostasis in apple. The figure is created with BioGDP.com [77]. Ca^2+^, calcium ion; CPC, CAPRICE; CPK, CALCIUM-DEPENDENT PROTEIN KINASE; GL2, GLABRA2; HAK1, HIGH-AFFINITY K^+^ TRANSPORTER 1; K^+^, potassium ion; Na^+^, sodium ion; OsSIT1, SALT-INDUCED RECEPTOR-LIKE KINASE 1; OsSERF1, SALT-RESPONSIVE ETHYLENE RESPONSE FACTOR 1; OsIPA1, IDEAL PLANT ARCHITECTURE 1; OsDREB2A, DEHYDRATION-RESPONSIVE ELEMENT-BINDING PROTEIN 2A; OsZFP179, ZINC FINGER PROTEIN 179; SOS1, SALT OVERLY SENSITIVE 1; PG2, POLYGALACTURONASE 2; TTG1, TRANSPARENT TESTA GLABRA 1.

Soil salinization, driven by improper irrigation, fertilizer misuse, and pollution, affects nearly 7% of global arable land and reduces yields by ~20% in irrigated areas [78]. Salt stress disrupts osmotic balance, ion homeostasis, and redox stability, severely impacting plant growth and productivity [6, 11]. In this context, MAPK cascades serve as conserved signaling modules that translate salt-induced cellular perturbations into adaptive transcriptional and physiological responses, making them key targets for improving salt tolerance (Fig. 3; Table 3). Although the MAPK-mediated salt signaling network has been elucidated in model species, its complexity in horticultural and medicinal plants remains largely unresolved. It is still unclear how MAPK modules integrate phytohormone regulation, ROS detoxification, ion balance, and developmental adaptation, or how specific cascades confer lineage- and tissue-specific salt tolerance.

In Arabidopsis, the MAPKK2–MAPK4/6 module enhances salt tolerance by activating stress-responsive genes [79]. In rice, OsMAPK5 phosphorylates SALT-RESPONSIVE ETHYLENE RESPONSE FACTOR 1 (OsSERF1), thereby amplifying salt-responsive signaling [80]. Similarly, SALT-INDUCED RECEPTOR-LIKE KINASE 1 (OsSIT1) activates OsMAPK3/6, promoting ethylene biosynthesis and ROS accumulation [59], whereas OsMAPK4 negatively regulates salt tolerance by phosphorylating IDEAL PLANT ARCHITECTURE 1 (IPA1) [53]. Interestingly, CALCIUM-DEPENDENT PROTEIN KINASE 5/13 (OsCPK5/13) can directly activate OsMAPK3/6 independent of the canonical MAPKKs, further enhancing salt resilience [51]. Beyond model species, MAPK cascades are also crucial in horticultural and medicinal crops. In maize (*Zea mays*), ZmMAPK7 phosphorylates ZmWRKY104, which activates *SUPEROXIDE DISMUTASE 4* (*ZmSOD4*) expression, increasing superoxide dismutase activity and reducing ROS accumulation [81].

In the recretohalophyte *L. bicolor*, LbMAPK2 participates in salt gland development and ion secretion [37]. In cotton (*Gossypium hirsutum*), the GhMEKK3/8/31–GhMAPKK5–GhMAPK11/23 cascade enhances salt tolerance by modulating WRKY-dependent transcription and adjusting both ABA and proline metabolism [82]. In apple, MdMAPK3 phosphorylates MdWRKY17 to activate the membrane transporter HIGH-AFFINITY K^+^ TRANSPORTER 1 (MdHAK1), maintaining Na^+^/K^+^ homeostasis [83]. Similarly, in alfalfa (*M. sativa*), MsMAPK3 phosphorylates MsNAC73, releasing repression on *POLYGALACTURONASE 2* (*MsPG2*) and enhancing salt tolerance [23]. Meanwhile, the ZmMAPK3–ZmGRF1 module promotes maize growth by enhancing cell proliferation under salt stress [84]. Collectively, these studies reveal that MAPK cascades regulate plant salt tolerance through multilayered mechanisms encompassing phytohormone signaling, ROS detoxification, ion homeostasis, and developmental plasticity. The diversity of MAPK modules among species reflects both evolutionary conservation and adaptive specialization. Future studies should dissect MAPK–Ca^2+^–phytohormone crosstalk, identify direct MAPK substrates via phosphoproteomics, and characterize regulatory nodes using gene-editing approaches. Integrating transcriptomic, proteomic, and physiological analyses in horticultural and medicinal plants will clarify how MAPK diversification drives salt stress adaptation and support the breeding of salt-tolerant cultivars.

### MAPK-mediated coordination of heat and cold signaling networks enhances plant thermotolerance

**Figure 4:**
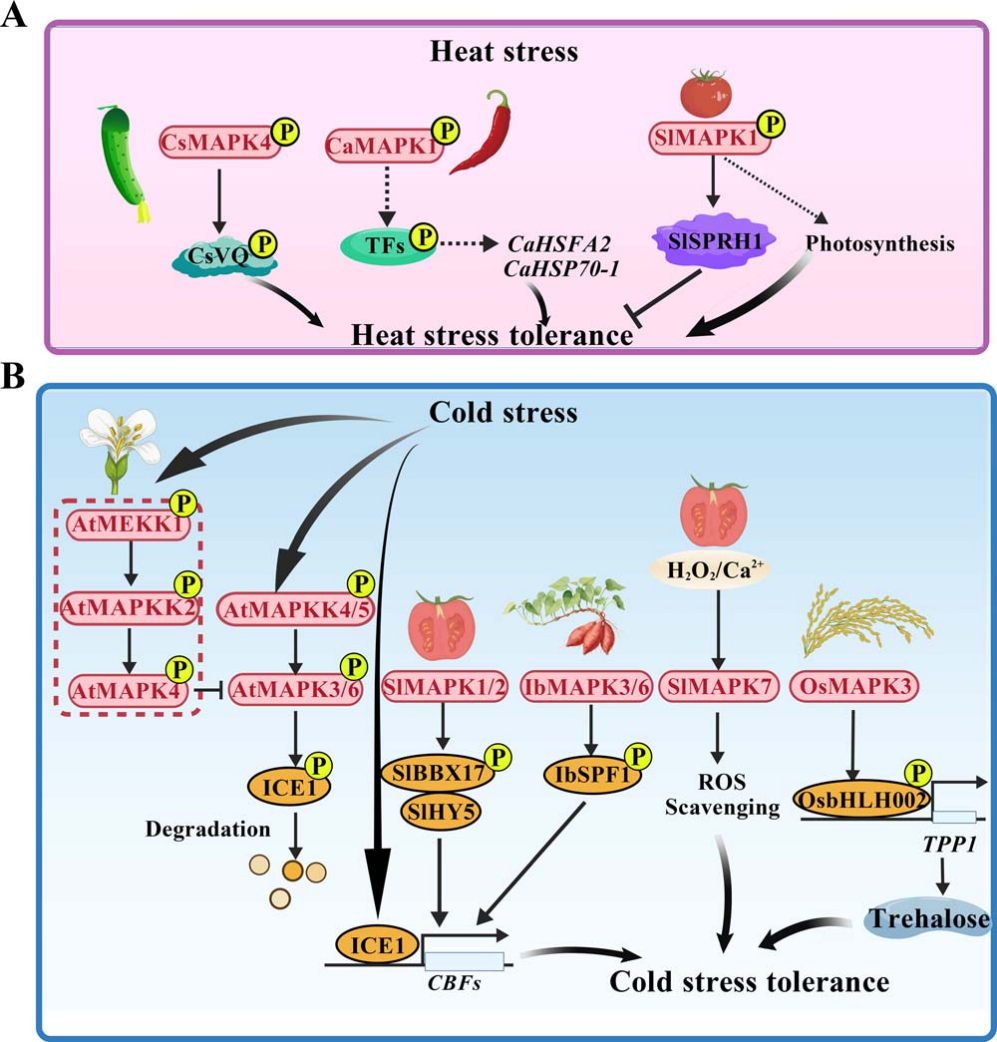
MAPK-mediated regulation of plant tolerance to extreme temperature stress. **(A)** In cucumber, CsMAPK4 phosphorylates CsVQ10 to enhance heat tolerance; in pepper, CaMAPK1 activates *CaHSFA2* and *CaHSP70–1* through phytohormone and ROS signaling; and in tomato, SlMAPK1 phosphorylates SlSPRH1 to modulate photosynthesis and antioxidant defense. **(B)** Under cold stress, MAPK cascades converge on the ICE–CBF module. In Arabidopsis, the AtMAPKK4/5–AtMAPK3/6 pathway destabilizes ICE1, whereas the AtMEKK1–AtMAPKK2–AtMAPK4 cascade enhances freezing tolerance. In tomato, SlMAPK1/2 phosphorylates SlBBX17 to promote SlHY5-dependent CBF activation, while SlMAPK7 improves ROS scavenging via H₂O₂/Ca^2+^ signaling. In rice, OsMAPK3 phosphorylates OsbHLH002 to activate *OsTPP1* and trehalose biosynthesis; in sweet potato, IbMAPK3/6 phosphorylates IbSPF1 to enhance photosynthesis and membrane stability. The figure is created using BioGDP.com [77]. CaHSFA2, HEAT SHOCK TRANSCRIPTION FACTOR A2; CaHSP70–1, HEAT SHOCK PROTEIN 70–1; CBF, C-REPEAT BINDING FACTOR; CsVQ10, VALINE-GLUTAMINE MOTIF PROTEIN 10; HSF, HEAT SHOCK TRANSCRIPTION FACTOR; HSP, HEAT SHOCK PROTEINS; IbSPF1, STRESS-RESPONSIVE TRANSCRIPTION FACTOR 1; ICE, INDUCER OF CBF EXPRESSION; OsTPP1, TREHALOSE-6-PHOSPHATE PHOSPHATASE 1; SlSPRH1, SERINE–PROLINE-RICH PROTEIN HOMOLOG 1; SlBBX17, B-BOX PROTEIN 17; SlHY5, ELONGATED HYPOCOTYL 5; TPP1, TREHALOSE-6-PHOSPHATE PHOSPHATASE 1.

Temperature is a critical environmental determinant of plant growth and productivity. Both heat and cold extremes disrupt membrane integrity, protein homeostasis, and metabolic balance, ultimately leading to yield losses [6, 11]. MAPK cascades act as conserved signaling hubs that perceive temperature fluctuations and orchestrate downstream transcriptional and physiological adjustments to maintain cellular homeostasis (Fig. 4; Table 3). Despite significant advances in model species, the molecular coordination of MAPK-mediated heat and cold signaling remains poorly defined, particularly in horticultural and medicinal plants. It is unclear how distinct MAPK modules exert either antagonistic or synergistic roles under extreme temperatures, or how they balance growth–defense trade-offs during thermal adaptation.

Under heat stress, MAPKs regulate thermotolerance through modulation of heat shock proteins, antioxidant defenses, photosynthesis, and developmental transitions (Fig. 4A). In cucumber (*Cucumis sativus*), CsMAPK4 interacts with VALINE-GLUTAMINE 10 (CsVQ10) as a phosphorylation substrate to positively regulate heat tolerance [85]. In pepper (*Capsicum annuum*), *CaMAPK1* is induced by high temperature and multiple phytohormones (SA, JA, ABA), as well as *Ralstonia solanacearum* infection; silencing CaMAPK1 represses *HEAT SHOCK TRANSCRIPTION FACTOR A2* (*CaHSFA2*) and *CaHSP70–1*, compromising thermotolerance and phytohormone/ROS signaling [86]. In tomato, proteomic analysis revealed that interference of SlMAPK1 alters the abundance of photosynthesis-related proteins, enhancing photosynthetic efficiency in knockdown plants—indicating a negative regulatory role [87]. Mechanistically, heat-activated SlMAPK1 phosphorylates SERINE-PROLINE-RICH PROTEIN HOMOLOG 1 (SlSPRH1), fine-tuning antioxidant defenses as a negative regulator of heat tolerance [88]. In lettuce (*Lactuca sativa*), silencing *LsMAPK4* markedly suppresses heat-induced bolting, confirming its positive role in heat-promoted flowering [89]. In Arabidopsis, MAPK-mediated phosphorylation of AUXIN/INDOLE-3-ACETIC ACID (AtIAA8) inhibits floral development under high temperature, indicating a conserved mechanism for developmental control under thermal stress [90].

Cold signaling through MAPKs converges on the C-REPEAT BINDING FACTOR/INDUCER OF CBF EXPRESSION (CBF/ICE) module (Fig. 4B). In Arabidopsis, the AtMAPKK4/5–MAPK3/6 pathway phosphorylates and promotes degradation of AtICE1, thereby repressing CBF-mediated freezing tolerance [91, 92]. Conversely, the AtMEKK1–MAPKK2–MAPK4 module enhances cold tolerance by suppressing AtMAPK3/6 activity [91]. In tomato, SlMAPK1/2 phosphorylates B-BOX CONTAINING PROTEIN 17 (SlBBX17), strengthening its interaction with ELONGATED HYPOCOTYL 5 (SlHY5) to activate CBF-dependent acclimation [93]. Moreover, *SlMAPK7*, a cold-induced MAPK activated by H_2_O_2_ and Ca^2+^, enhances chilling tolerance via reducing ROS accumulation, elevating antioxidant enzyme activity, and promoting proline and soluble sugar accumulation [94]. In rice, OsMAPK3 interacts with the TF OsbHLH002, preventing its degradation by HIGH EXPRESSION OF OSMOTICALLY RESPONSIVE GENES 1 (OsHOS1) and phosphorylating it to enhance transactivation of *TREHALOSE-6-PHOSPHATE PHOSPHATASE 1* (*OsTPP1*), leading to trehalose accumulation and improved cold tolerance [33]. In sweet potato (*Ipomoea batatas*), IbMAPK3/IbMAPK6 phosphorylate and stabilize STRESS-RESPONSIVE TRANSCRIPTION FACTOR 1 (IbSPF1), enhancing photosynthesis and reducing lipid peroxidation under cold stress [95].

MAPK cascades function as multifaceted regulators of plant thermotolerance. Under heat stress, MAPKs control the expression of heat shock proteins, antioxidant enzymes, photosynthetic activity, and developmental transitions such as flowering. Under cold stress, MAPKs enhance tolerance primarily via the CBF/ICE module, osmolyte accumulation (trehalose, proline), and antioxidant activation. The coordinated yet sometimes opposing activities of distinct MAPK modules form a dynamic signaling network that enables plants to withstand thermal extremes. Future research should focus on elucidating the crosstalk between heat- and cold-activated MAPK modules, identifying their phosphorylation substrates, and clarifying how MAPKs integrate temperature sensing with phytohormone and metabolic responses. Functional genomics and phosphoproteomic approaches in horticultural and medicinal plants will be essential for understanding MAPK diversification and for developing thermotolerant cultivars.

## MAPK regulates secondary metabolite biosynthesis

Understanding the regulatory roles of MAPK cascades in secondary metabolism is of great theoretical and practical importance for improving the quality, stress resistance, and bioactive compound production in horticultural and medicinal plants [2–4]. Under stress conditions, dynamic changes in signaling metabolites and TFs drive metabolic reprogramming [12]. As a central signaling hub, MAPK cascades play a pivotal role in coordinating the biosynthesis of phenylpropanoids, terpenoids, alkaloids, and other secondary metabolites. Despite advances in model species, key questions remain for horticultural and medicinal plants: how do distinct MAPK modules decode specific cues (temperature, light, drought, salt, and phosphate deficiency) to direct branch-specific flux? How does MAPK-dependent phosphorylation modulate TF activity and the assembly of MYB/WRKY/bHLH/ERF complexes, and adjust the stability of structural enzymes? To what extent are these MAPK–TF–enzyme circuits conserved versus lineage-specific?

### Pigmentation and quality-related metabolites in horticultural plants

**Figure 5:**
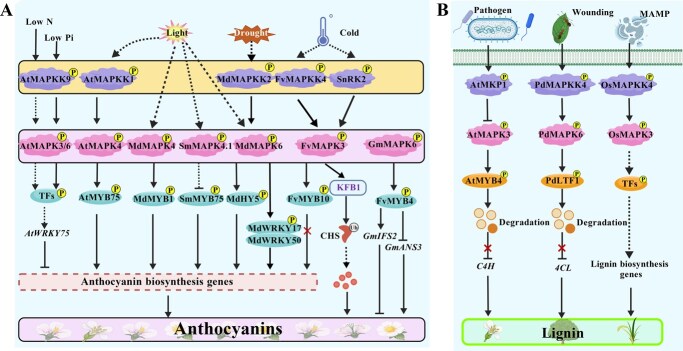
MAPK integrates environmental signals to regulate the biosynthesis of anthocyanin and lignin in horticultural plants. **(A)** MAPKs integrate environmental and nutritional cues such as light, drought, temperature, and nutrient deficiency to fine-tune pigment biosynthesis. In apple, MdMAPK6–MdHY5 and MdMAPK4–MdMYB1 promote light- and drought-induced anthocyanin accumulation. In Arabidopsis, light-activated AtMAPK4 phosphorylates AtMYB75, while the AtMAPKK9–AtMAPK3/6 cascade under low phosphate or nitrogen represses anthocyanin synthesis. FvMAPK3 in strawberry and SmMAPK4.1 in eggplant negatively regulate pigmentation by targeting FvMYB10 and SmMYB75, respectively. In soybean, GmMAPK6–GmMYB4 modulates the MBW complex to shift metabolic flux toward isoflavone rather than anthocyanin biosynthesis. **(B)** MAPKs also regulate lignin formation under stress. In Arabidopsis, AtMKP1 relieves AtMYB4-mediated repression of lignin genes. In poplar, PdMAPK6 phosphorylates PdLTF1 to promote its degradation and activate *4CL*. In rice, the OsMAPKK4–OsMAPK3/6 pathway redirects carbon flux to lignin and phytoalexin synthesis upon chitin stimulation. The figure is created using BioGDP.com [77]. 4CL, 4-COUMARATE: COA LIGASE; ANS, ANTHOCYANIDIN SYNTHASE; C4H, CINNAMATE-4-HYDROXYLASE; IFS, ISOFLAVONE SYNTHASE; MAMP, microbe-associated molecular pattern; PdLTF1, LIGNIN BIOSYNTHESIS-ASSOCIATED TRANSCRIPTION FACTOR 1.

Evidence across multiple species indicates that MAPKs fine-tune pigment metabolism by post-translationally modifying key TFs and structural enzymes, thereby integrating environmental cues such as light, temperature, drought, and phosphate deficiency into pigment biosynthesis (Fig. 5A; Table 4) [12, 96]. Notably, in apple, light signaling activates two major axes, MdMAPK6–MdHY5 and MdMAPK4–MdMYB1, where MdMAPK6 phosphorylates and stabilizes MdHY5 to promote light-induced anthocyanin accumulation [97], while MdMAPK4 enhances MdMYB1 stability and coloration [98]. Similarly, in Arabidopsis, light-activated AtMAPK4 phosphorylates AtMYB75, which is essential for anthocyanin biosynthesis [29]. This regulation exhibits both activating and repressing patterns [27, 30]. Moreover, under phosphate- and nitrogen-deficient conditions, the AtMAPKK9–MAPK3/6 cascade promotes the expression of *AtWRKY75* and represses the biosynthesis of anthocyanins by phosphorylating an unidentified TF in Arabidopsis [27, 30]. In contrast, in strawberry, low temperature induces *FvMAPK3*, which suppresses anthocyanin accumulation by phosphorylating FvMYB10 to reduce its transcriptional activity and CHALCONE SYNTHASE 1 (FvCHS1) to promote its proteasomal degradation [99]. In eggplant, SmMAPK4.1 interacts with SmMYB75 to negatively regulate high light-induced pigmentation, while knockout of *SmMAPK4.1* alleviates this inhibition [38]. In drought-stressed apple, the MdMEK2–MdMAPK6–MdWRKY17–MdWRKY50 cascade enhances anthocyanin accumulation and oxidative stress tolerance by activating *MdCHS* [100]. Additionally, dark-induced MdMAPK4 phosphorylates ETHYLENE RESPONSE FACTOR 17 (MdERF17) to promote chlorophyll degradation during light/dark transitions, coordinating spatiotemporal pigmentation [98]. In soybean (*Glycine max*), the GmMAPK6–GmMYB4–MBW module reallocates flux between isoflavone and anthocyanin branches: GmMAPK6 phosphorylates and activates GmMYB4, and GmMYB4 modulates MYB–bHLH–WD40 (MBW) complex assembly to differentially regulate I*SOFLAVONE SYNTHASE 2* (*GmIFS2*) and *ANTHOCYANIDIN SYNTHASE 3* (*GmANS3*), increasing isoflavones while suppressing anthocyanins [101].

**Table 4:** MAPKs are involved in the regulation of plant secondary metabolites

**Category**	**Secondary metabolites**	**Stimuli**	**Species**	**MAPK**	**Substrates/Pathways**	**Reference**
Pigmentation and quality-related metabolites	Anthocyanins	N/Pi-insufficient	*A. thaliana*	AtMAPK3/6	AtMAPKK9-MAPK3/6-WRKY75	[27, 30]
	Anthocyanins	Light	*A. thaliana*	AtMAPK4	AtMYB75	[29]
	Anthocyanins	Low temperature	*F. vesca*	FvMAPK3	FvMYB10	[99]
	Anthocyanins	Light	*M. domestica*	MdMAPK4	MdMYB1	[98]
	Anthocyanins	Light	*M. domestica*	MdMAPK6	MdHY5	[97]
	Anthocyanins	Drought	*M. domestica*	MdMAPK6	WRKY17	[100]
	Anthocyanins	Light	*S. melongena*	SmMAPK4.1	SmMYB75	[38]
	Anthocyanins/ Isoflavones	Unidentified	*Glycine max*	GmMAPK6	GmMYB4	[101]
	Lignin	Pathogen	*A. thaliana*	AtMAPK3/6	AtMKP1-MAPK3/6-MYB4	[102]
	Lignin	Chitin	*O. sativa*	OsMAPK3	OsMAPKK4-MAPK3	[25]
	Lignin	Wounding	*Populus dicot*	PdMAPK6	PdMAPKK4-MAPK6-LTF1	[103]
Medicinally active components	Crocins	JA	*Crocus sativus*	CsMAPK6	CsMAPK6-WRKY38/34	[109]
	Diterpenoid/Linalool	*M. oryzae*	*O. sativa*	OsMAPK3	OsBDR1–MAPK3-TPS3/29	[34]
	Forsythin	Unidentified	*F. suspensa*	FsMAPK3	FsWRKY4	[107]
	Salvianolic acids	JA/SA	*S. miltiorrhiza*	SmMAPK3	SmJAZs	[105]
	Salvianolic acids	SA	*S. miltiorrhiza*	SmMAPK3	SmRAS1	[104]
	Tanshinones	JA	*S. miltiorrhiza*	SmMAPK3	SmWRKY33	[106]
	Terpenoids	Unidentified	*B. platyphylla*	BpMAPK6	BpWRKY6	[108]
Defense-related metabolites	Glucosinolates/ Camalexin	*Botrytis cinerea*	*A. thaliana*	AtMAPK3/6	AtMAPKKK3/5-MAPKK4/5 -MAPK3/6-WRKY33	[48, 51, 114]
	Monoterpenoid indole alkaloids	UV-B/JA	*C. roseus*	CrMAPK3	Unidentified	[115, 116]
	Nicotine	JA	*N. tabacum*	NtMAPK4	NtMEKK1b-MAPKK2a- MAPK4-ERF221	[117, 118]
	Phytoalexins	Pathogen	*O. sativa*	OsMAPK3	OsNAC29	[112]
	Phytoalexins	Chitin	*O. sativa*	OsMAPK6	OsMAPKK4-MAPK6- OsVQ8-WRKY10	[113]
	Phytoalexins	Chitin	*O. sativa*	OsMAPK3/6	OsMAPKK4-MAPK3/6	[25]
	Sakuranetin	Pathogen	*O. sativa*	OsMAPK6	OsWRKY67	[111]

MAPKs are also involved in lignin biosynthesis (Fig. 5B; Table 4). In Arabidopsis, MAPK PHOSPHATASE 1 (MKP1) regulates lignin biosynthesis by controlling MAPK-mediated phosphorylation of MYB4, thereby relieving its repression and promoting vascular lignification and disease resistance [102]. In rice, the fungal-type microbial-associated molecular pattern (MAMP) chitin activates the OsMAPKK4–OsMAPK3 cascade to reprogram defense metabolism. Activated OsMAPKK4 further redirects carbon flux from glycolysis toward secondary metabolism and induces cell death as well as diterpenoid and lignin synthesis [25]. Similarly, in poplar, phosphorylation of LIGNIN BIOSYNTHESIS-ASSOCIATED TRANSCRIPTION FACTOR 1 (PdLTF1) by PdMAPK6 promotes its proteasomal degradation, relieving repression on lignin biosynthetic gene *4-COUMARATE: COA LIGASE* (*4CL*) and initiating lignification under stress [103].

### Bioactive metabolites in medicinal plants

**Figure 6:**
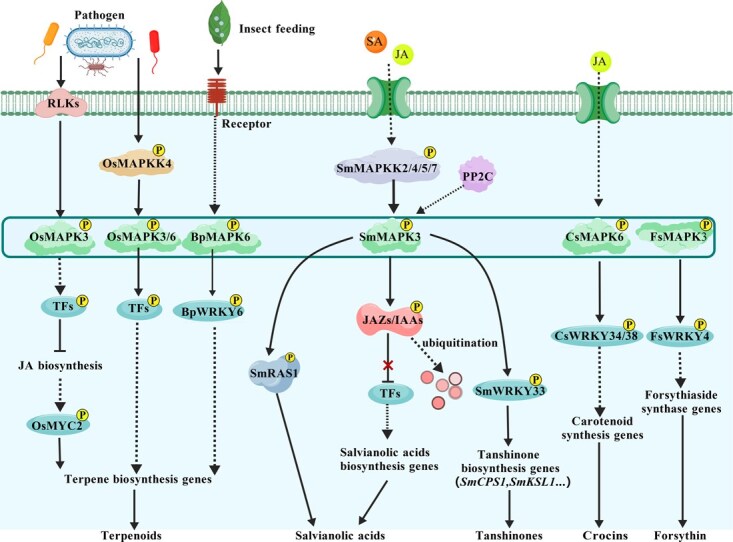
MAPK-mediated regulation of bioactive metabolite biosynthesis in medicinal plants. MAPK cascades connect environmental and hormonal signals with the biosynthesis of pharmacologically active compounds in medicinal plants. In *S. miltiorrhiza*, SmMAPK3 acts as a central regulator in two phosphorylation modules: (i) the SA–SmMAPK3–SmRAS1 axis, which promotes salvianolic acid accumulation via phosphorylation-induced stabilization of SmRAS1, and (ii) the SmMAPKK2/4/5/7–SmMAPK3–SmWRKY33 module, which activates tanshinone biosynthetic genes by relieving JA-mediated repression. Similar MAPK–WRKY signaling frameworks control specialized metabolism in other medicinal plants: FsMAPK3–FsWRKY4 in *F. suspensa* (forsythin biosynthesis), BpMAPK6–BpWRKY6 in birch (terpene synthesis), and CsMAPK6–CsWRKY34/38 in saffron (crocin biosynthesis). In rice, the RLK OsBDR1–MAPK3 module regulates terpenoid synthesis during *M. oryzae* infection. The figure is created using BioGDP.com [77]. RLKs, RECEPTOR-LIKE KINASES; SmRAS1, ROSMARINIC ACID SYNTHASE 1; SmCPS1, COPALYL DIPHOSPHATE SYNTHASE 1; SmKSL1, KAURENE SYNTHASE-LIKE 1.

As a model medicinal plant, *S. miltiorrhiza* produces two major classes of pharmacologically active compounds—lipophilic tanshinones and hydrophilic phenolic acids [42, 104]. MAPK cascades serve as a central link between environmental stimuli and these biosynthetic pathways. SmMAPK3 functions as the core regulator in two phytohormone-dependent phosphorylation modules (Fig. 6; Table 4): (i) the SA–SmMAPK3**–**ROSMARINIC ACID SYNTHASE 1 (SmRAS1) axis, where SA-induced phosphorylation of SmRAS1 enhances its stability and activity, promoting phenolic acid accumulation [104, 105]; and (ii) the SmMAPKK2/4/5/7–SmMAPK3–SmWRKY33 axis, which enhances tanshinone biosynthesis by alleviating JA-mediated repression and activating downstream tanshinone biosynthetic genes [106]. Similar MAPK–WRKY modules have been characterized in other medicinal plants. In *Forsythia suspensa*, the FsMAPK3–FsWRKY4 module likely regulates forsythin biosynthesis [107]; in birch, BpMAPK6–BpWRKY6 activates JA and terpene synthesis, enhancing insect resistance [108]; In saffron, JA activates the CsMAPK6–CsWRKY34/38 module, which coordinately regulates carotenoid and apocarotenoid biosynthesis, leading to enhanced crocin accumulation [109]. Similarly, during *Magnaporthe oryzae* infection in rice, the RECEPTOR-LIKE KINASE (RLK) BLAST DISEASE RESISTANCE 1 (OsBDR1) phosphorylates OsMAPK3, thereby upregulating the expression of *TERPENE SYNTHASE 3* (*OsTPS3*) and *OsTPS29*, and promoting the synthesis of diterpenoids and linalool [34]. In addition, in salt-tolerant peppermint *(Mentha piperita*), MAPK activation modulates essential oil metabolism dynamically, maintaining the menthol/menthone ratio and ensuring stable productivity under salt stress conditions [110].

### Defensive metabolites

**Figure 7:**
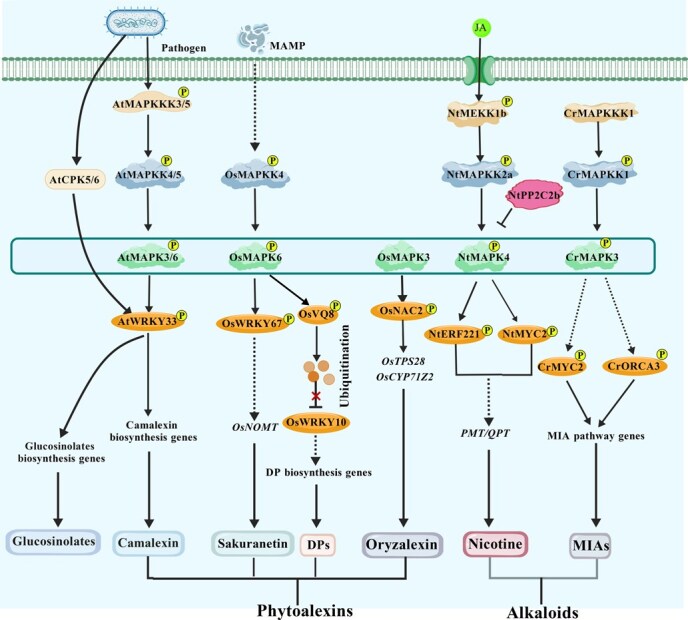
MAPK is involved in the biosynthesis of defensive metabolites such as alkaloids, glucosinolates, and phytoalexins. MAPK cascades integrate pathogen and JA signaling to regulate the biosynthesis of key defensive metabolites, including phytoalexins, glucosinolates, and alkaloids. The *Os*MAPK6–OsWRKY67–OsNOMT axis promotes sakuranetin accumulation, while OsMAPK3–OsNAC2 activates *OsTPS28* and *OsCYP71Z2* to enhance oryzalexin production. Upon pathogen perception, the activated OsMAPKK4*–*OsMAPK6 module phosphorylates OsVQ8, which interacts with OsWRKY10 to fine-tune diterpenoid phytoalexin biosynthesis. In Arabidopsis, both CPK5/6 and MAPK3/6 phosphorylate AtWRKY33, activating indolic glucosinolate and camalexin pathways. In *C. roseus*, the CrMAPKKK1–CrMAPKK1–CrMAPK3 cascade may phosphorylate CrMYC2 and CrORCA3, activating MIA biosynthetic genes. In *N. tabacum*, JA-induced NtMAPK4 forms a kinase–phosphatase module with NtPP2C2b to regulate ERF-dependent nicotine biosynthesis, ensuring balanced defense and metabolic output. The figure is created using BioGDP.com [77]. CrORCA3, OCTADECANOID-RESPONSIVE CATHARANTHUS AP2/ERF DOMAIN 3; DPs, diterpenoid phytoalexins; MIA, monoterpenoid indole alkaloid; NtERF221, ETHYLENE RESPONSE FACTOR 221; OsVQ8, VALINE–GLUTAMINE MOTIF-CONTAINING PROTEIN; OsNOMT, naringenin 7-O-methyltransferase; OsCYP71Z2, CYTOCHROME P450 FAMILY 71 SUBFAMILY Z POLYPEPTIDE 2.

Defensive metabolites such as diterpenoid phytoalexins, flavonoids, and alkaloids are crucial components of plant innate immunity [13, 47]. In rice, the OsMAPKK4–OsMAPK3/6 cascade channels the MAMP signals toward diterpenoid phytoalexins [25]. The OsMAPK6–OsWRKY67–NARINGENIN 7-O-METHYLTRANSFERASE (OsNOMT) axis promotes sakuranetin production and resistance to false smut [111], while OsMAPK3–OsNAC29 phosphorylates and stabilizes OsNAC29 to activate *OsTPS28* and *CYTOCHROME P450 FAMILY 71 SUBFAMILY Z POLYPEPTIDE 2* (*OsCYP71Z2*), enhancing oryzalexin accumulation [112]. Additionally, OsWRKY10 interacts with OsVQ8, and upon pathogen-associated molecular patterns (PAMPs) perception, the activated OsMAPKK4–OsMAPK6 cascade phosphorylates OsVQ8, which in turn modulates diterpenoid phytoalexin (DP) biosynthesis [113]. In Arabidopsis, both the CPK5/CPK6 and MAPK3/MAPK6 signaling pathways promote the biosynthesis of indolic glucosinolates and camalexin during defense against *Botrytis cinerea* [48]. Infection by *B. cinerea* activates AtCPK5/CPK6 and AtMAPK3/6; these kinases cooperatively phosphorylate AtWRKY33, thereby enhancing its transcriptional activation of camalexin biosynthetic genes and boosting camalexin production [48, 51]. In parallel, a canonical AtMAPKKK3/5–AtMAPKK4/5–AtMAPK3/6–AtWRKY33 cascade also contributes to the regulation of camalexin biosynthesis [48, 51, 114] (Fig. 7; Table 4).

Among defense metabolites, alkaloids represent a primary chemical barrier due to their bitterness, toxicity, and antimicrobial activity. In *Catharanthus roseus*, the CrMAPKKK1–CrMAPKK1–CrMAPK3/6 cascade may phosphorylate CrMYC2 and OCTADECANOID-RESPONSIVE CATHARANTHUS AP2/ERF DOMAIN 3 (CrORCA3), enhancing transcriptional activation of monoterpenoid indole alkaloid (MIA) pathway genes and increasing vinblastine and vincristine accumulation [115, 116]. In *Nicotiana tabacum*, JA-induced NtMAPK4 interacts with PHOSPHATASE TYPE 2C B (NtPP2C2b), forming a ‘kinase–phosphatase’ regulatory module that fine-tunes AP2/ERF TF and nicotine biosynthesis [117, 118]. Thus, MAPK and PP2C modules precisely couple JA signaling with alkaloid biosynthesis through post-translational regulation of transcriptional hubs, providing actionable molecular targets for metabolic engineering (Fig. 7; Table 4).

Together, MAPK cascades form integrative nodes that couple environmental/phytohormone inputs to TFs (WRKY/MYB/bHLH/ERF) and structural enzymes (CHS, RAS, CYPs), orchestrating dynamic rewiring of pigment, pharmacological, and defensive pathways. The architecture is broadly conserved (MAPK–TF–pathway gene chains) yet exhibits species- and pathway-specific specialization. Priorities include defining stimulus specificity and crosstalk with photoreceptors/thermosensors/phytohormones; mapping direct phospho-targets via phosphoproteomics and interactomics; and leveraging these modules for metabolic engineering in horticultural and medicinal plants to rationally steer flux and enhance quality traits.

## Conclusions

MAPK cascades are evolutionarily conserved switchboards that translate environmental and developmental cues into targeted transcriptional and metabolic outputs [8, 47]. In horticultural and medicinal plants—where yield, quality, and pharmacological value depend on both stress resilience and specialized metabolites—MAPKs couple abiotic stress tolerance (drought, salt, extreme temperature) with the regulation of phenylpropanoid, terpenoid, alkaloid, and N and S-containing pathways that determine coloration, flavor, defense, and bioactivity [8, 11, 47]. Across model, horticultural, and medicinal plants such as Arabidopsis [58], rice [119], tomato [69, 71, 120], and apple [83], converging evidence highlights MAPKs as pivotal regulators of abiotic stress tolerance. Under drought, salinity, and temperature extremes, MAPK cascades integrate upstream stress perception with downstream responses by modulating ABA-, JA-, SA-, and ET-dependent signaling pathways [59, 65, 69]. Through phosphorylation of TFs and antioxidant enzymes, MAPKs enhance ROS scavenging, adjust stomatal and osmotic balance, and stabilize cellular homeostasis to promote stress resilience. Beyond stress responses, MAPK modules also fine-tune the biosynthesis of secondary metabolites that determine pigmentation, flavor, defense, and pharmacological activity. Environmental and biotic cues—such as light, temperature, and pathogen attack—activate MAPKs, which in turn phosphorylate key TFs such as WRKY, MYB, bHLH, and ERF, and metabolic enzymes (e.g. CHS, CYPs, RAS). This regulation shapes metabolic flux through phenylpropanoid, terpenoid, alkaloid, and N and S-containing pathways. Together, these findings establish MAPKs as dual hubs that couple environmental adaptation with metabolic specialization, offering an integrated systems framework for understanding and improving stress tolerance, coloration, and bioactive compound accumulation in horticultural and medicinal crops.

## Perspectives

Despite substantial progress in elucidating MAPK signaling, our understanding of its regulatory logic in horticultural and medicinal plants remains fragmentary. Current research is disproportionately focused on models and a few horticultural species under controlled conditions, while medicinal plants, field-like combined stresses, and spatial or temporal regulation remain underexplored. Functional studies are largely confined to Arabidopsis MAPK3/4/6, leaving the roles, hierarchies, and cross-species conservation of other MAPK members unresolved. The causal chain ‘MAPK-substrate-metabolic flux-phenotype’ also remains incomplete. Moreover, MAPKs rarely function alone: many substrates are cophosphorylated by other kinases such as CPKs or SnRK2s, creating complex signaling hierarchies whose coordination principles are still poorly defined [48, 51, 76]. Crosstalk with other PTMs (e.g. SUMOylation and ubiquitination) further shapes protein stability and activity, yet the combinatorial logic governing these interactions remains obscure [112, 121, 122]. Additionally, the constitutive activation of MAPK pathways often enhances stress tolerance at the cost of growth and productivity, highlighting the persistent challenge of mitigating growth–defense trade-offs in applied contexts.

Future research should therefore pursue integrative, multiscale, and context-dependent approaches to decode MAPK regulation and its translational potential. Systematic phenotyping and functional genomics—using CRISPR editing, inducible knockouts, and phosphoproteomic mapping—should be applied across representative species such as tomato, apple, and *S. miltiorrhiza* to resolve the dynamic relationships between MAPK modules, substrates, and metabolic outputs. Advanced multiomics combined with interpretable machine learning can further delineate stress-responsive network rewiring under realistic environmental conditions. Meanwhile, chemical ecology and chemical biology approaches may uncover natural microbial and rhizosphere metabolites (e.g. ‘albaflavenone-like’ activators) that fine-tune MAPK signaling, offering sustainable strategies for stress priming. Synthetic biology provides another frontier: engineering tissue- and stage-specific promoters, environmental switches, and modular ‘MAPK–TF–enzyme’ circuits could enable precise spatiotemporal activation of MAPK cascades, balancing defense with growth. Ultimately, integrating kinase cooperation, PTM crosstalk, and synthetic control will transform MAPK from passive signal relays into programmable regulatory nodes—driving coordinated improvements in stress resilience, metabolic efficiency, and quality formation in horticultural and medicinal plants [123].

Finally, by consolidating functional evidence across model, horticultural, and medicinal plants, this review outlines an MAPK-centered regulatory architecture that connects abiotic (drought, salinity, temperature, light, phytohormones, nutrients) and biotic (pathogen attack, insect herbivory) signals to metabolic adaptation. In this framework, the MAPKKK–MAPKK–MAPK cascade funnels upstream cues into three coordinated regulatory arms: (i) phytohormone crosstalk involving ABA, JA, SA, and ET pathways; (ii) phosphorylation of transcription factors such as WRKY, MYB, bHLH, ERF, and bZIP; and (iii) modulation of structural enzymes including CHS, CYPs, and RAS. Together, these arms orchestrate stress tolerance and the redistribution of metabolic flux across phenylpropanoid, terpenoid, alkaloid, and N- and S-containing pathways, resulting in concurrent improvements in stress resilience and the biosynthesis of high-value functional compounds. In summary, viewing MAPKs not only as mechanistic hubs but also as programmable design nodes provides a practical strategy to integrate stress signaling with metabolic enhancement—laying a conceptual foundation for rational engineering and breeding of resilient, high-quality horticultural and medicinal plants.

## Supplementary Material

Web_Material_uhaf350
